# Recurrent Hypoglycemia in a Non-diabetic Female: A Case Study on Doege-Potter Syndrome

**DOI:** 10.7759/cureus.63329

**Published:** 2024-06-27

**Authors:** Saeed R Mohammed, Narine Mack, Valmikie Ramoutar, Jessica Rampersad

**Affiliations:** 1 Department of Clinical Medical Sciences, The University of the West Indies, St. Augustine Campus, Champs Fleurs, TTO; 2 Department of Medicine, San Fernando General Hospital, San Fernando, TTO

**Keywords:** solitary fibrous tumor, solitary fibrous pleural tumour, endocrinology, recurrent hypoglycemia, hypoglycemia

## Abstract

Solitary fibrous tumors (SFT) are mesenchymal cell tumors that may arise from any site throughout the body. A small percentage of patients with SFT develop non-islet cell tumor-induced hypoglycemia (NICTH), eponymously termed Doege-Potter Syndrome (DPS). DPS is characterized by severe, refractory hypoinsulinemic hypoglycemia.

Diagnosis of SFT is dependent on histologic findings and immunohistochemistry (IHC). NAB2-STAT6 gene fusions are pathognomonic for SFT but may be difficult to identify in routine cytogenetic studies. STAT6 IHC is a highly sensitive and specific surrogate for the NAB2-STAT6 gene fusion.

Total resection of the tumor remains the gold-standard definitive treatment of SFT of the pleura. Palliative tumor debulking is recommended if total resection is not feasible.

We here report a case of DPS in a 73-year-old female, managed with palliative care.

## Introduction

Solitary fibrous tumors (SFT), first described by Wagner in 1870 [1], are mesenchymal cell tumors predominantly of pleural origin, but may arise from any site throughout the body [2,3]. SFT of the pleura (SFTP) accounts for ≈5% of all pleural neoplasms [4]. They typically occur in the sixth and seventh decades of life with similar incidences between the sexes [2]. They are most frequently benign and asymptomatic but may present with paraneoplastic syndromes in ≤20% of cases [5,6]. A small percentage of patients with SFT (≤5%) [6,7], develop non-islet cell tumor-induced hypoglycemia (NICTH); initially independently reported by Doege [8] and Potter [9] in 1930, hence the eponymous Doege-Potter Syndrome (DPS).

DPS is characterized by severe, refractory hypoinsulinemic hypoglycemia resulting from the secretion of a prohormone or incompletely processed molecule of insulin-like growth factor (IGF) II, first identified by Daughaday et al. in 1988 [10]. Meng et al. in their 2014 review reported a roughly equal number of cases between benign and malignant SFTP with DPS [6].

We here report a case of DPS in a 73-year-old female; her tumor was deemed unresectable, and she received palliative care.

## Case presentation

A 73-year-old female, with known primary hypothyroidism and a four-year history of spindle cell lung cancer presented to our institution with complaints of recurrent hypoglycemic episodes. She had presented on two occasions in the prior two weeks with complaints of generalized weakness, diaphoresis, and confusion, and had been managed symptomatically on each visit.

She was not known to have diabetes, nor did she have access to oral antidiabetic drugs or insulin. She reported a 20-pound weight loss over the preceding six months but denied appetite/dietary changes. She stated frequent/persistent episodes of hypoglycemic symptoms at home, which she self-managed by increased consumption of sugary snacks and drinks. She noted no consistent temporal association with her symptoms. She denied alcohol or glucocorticoid use and had no symptoms or stigmata of renal or liver disease.

She was admitted and continued to experience further episodes of spontaneous hypoglycemia, managed with intravenous (IV) 10% dextrose. Laboratory testing revealed normal renal and liver function. Synacthen test was normal (cortisol: 21.4 mg/dL measured 30 mins post 250 mcg synacthen IM).

A supervised fast was commenced and within four hours the patient developed hypoglycemia and random blood sugar (RBS) of 40 mg/dL. Concurrent results showed suppressed levels of insulin (<2.0) and C-Peptide (<2.0).

IGF testing was performed; IGF-I was decreased (20.4 ng/ml; normal range: 24-200 ng/ml) while IGF-II was elevated (419 ng/ml; normal range 333-967 ng/ml), i.e., an IGF-II:IGF-I ratio: 419:20.4. Institutional limitations rendered us unable to perform investigations for growth hormone, insulin antibodies, and antidiabetic drug screening.

**Figure 1:**
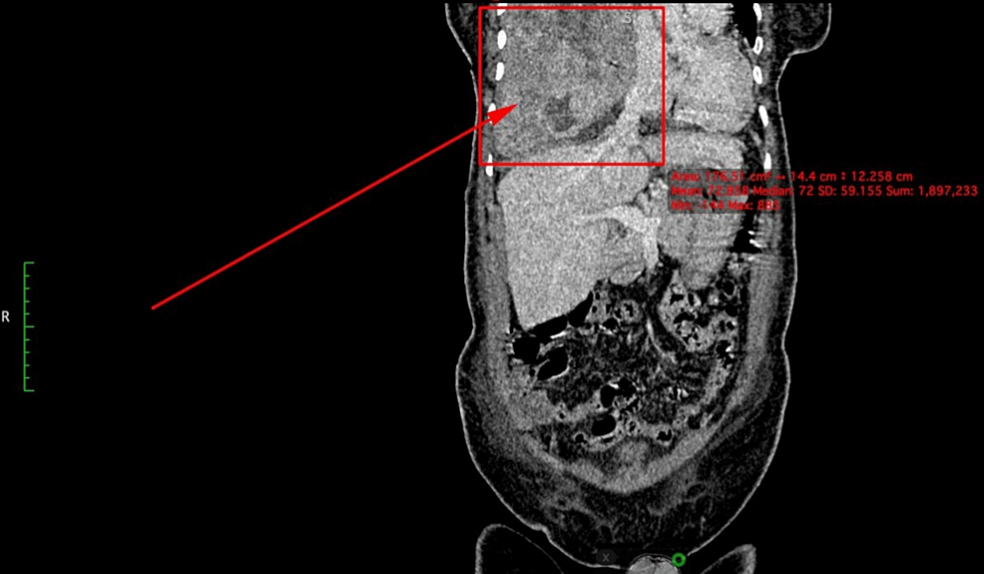
CT coronal IV contrast image demonstrates caudal effacement of the diaphragm with mass effect on the subdiaphragmatic structures such as the liver. CT: computed tomography; IV: intravenous

Computed tomography (CT) abdomen displayed caudal effacement of the diaphragm with mass effect on structurally normal subdiaphragmatic structures (Figure 1).

**Figure 2:**
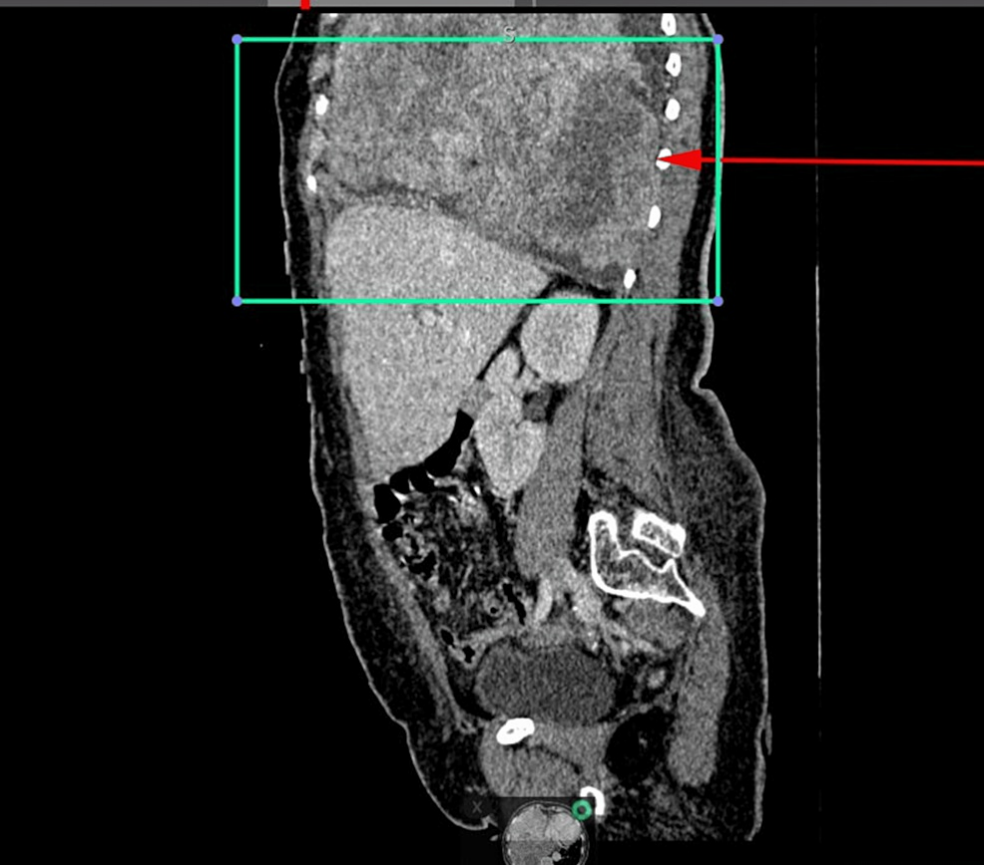
CT sagittal IV contrast demonstrates the large lesion limited by the diaphragm with no evidence of intraabdominal extension. CT: computed tomography; IV: intravenous

There was no evidence of intrabdominal extension (Figure 2).

**Figure 3:**
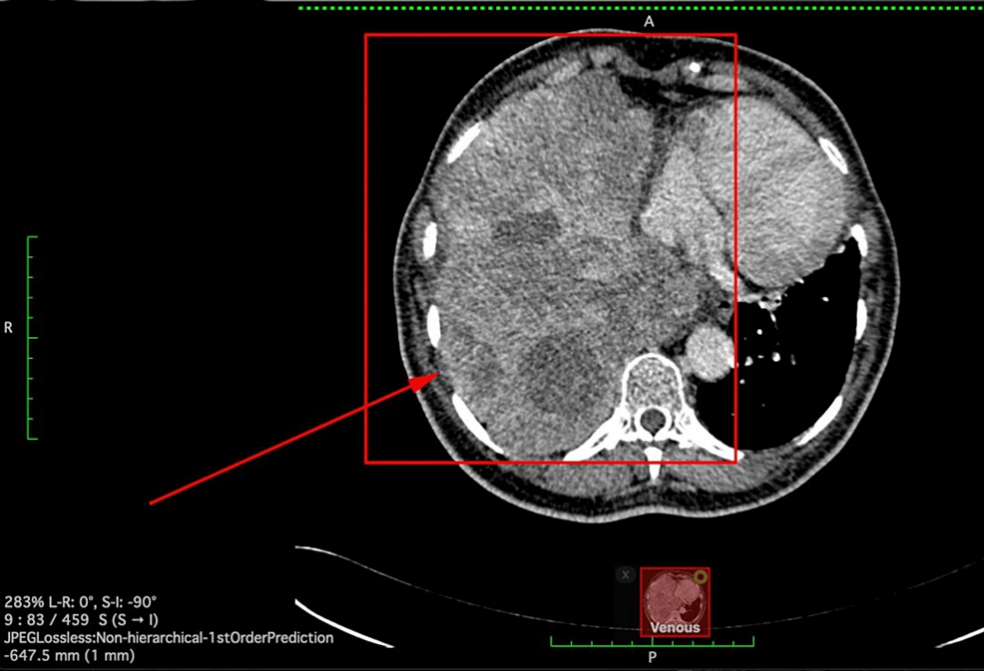
CT illustrating an axial image of a large infiltrative lesion occupying the right mid-lower lobe region of the hemithorax and displacement of the mediastinum to the left. CT: computed tomography

CT chest revealed a lobulated right lung mass measuring 17.6 cm x 12 cm x 10.5 cm (Figure 3).

**Figure 4:**
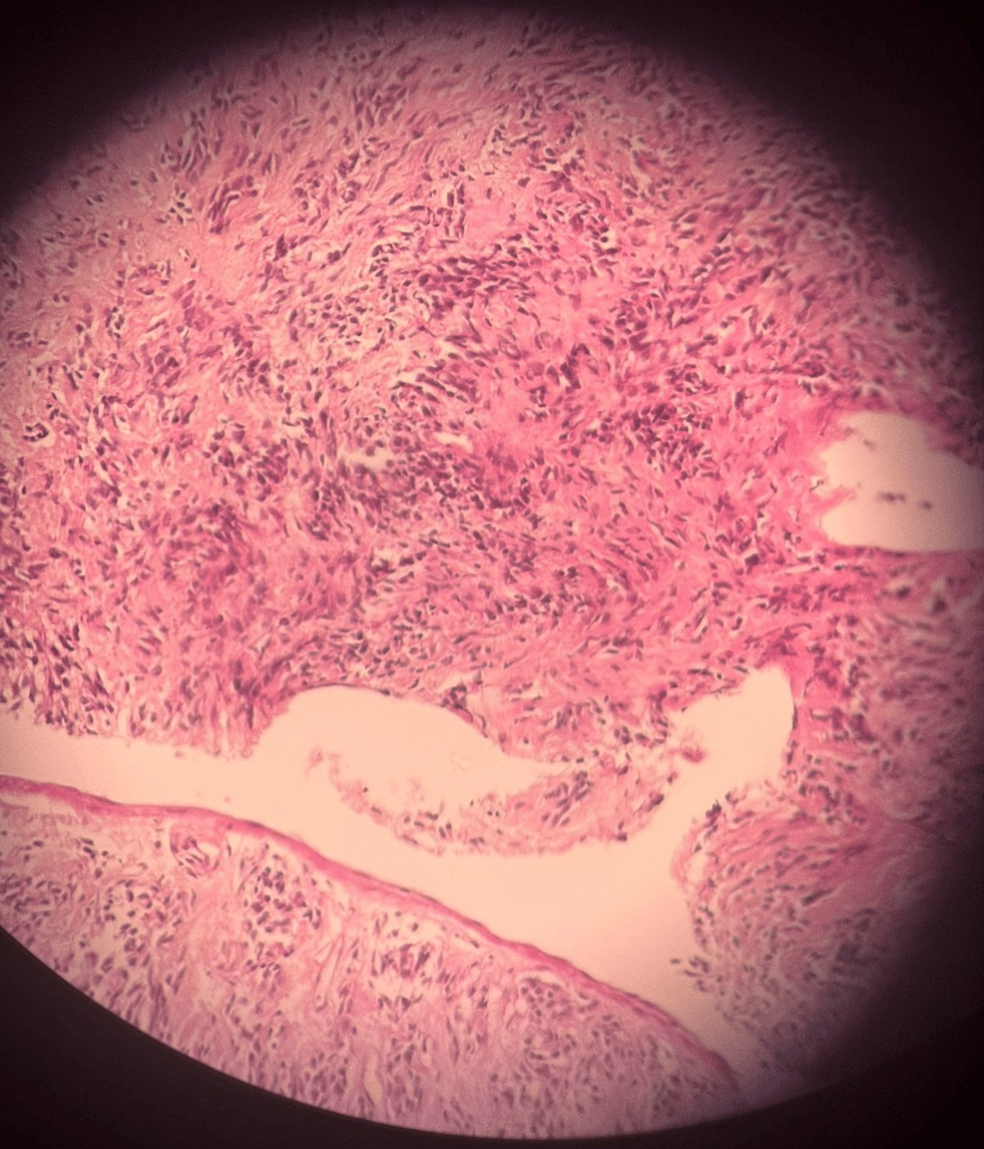
Lung biopsy specimen displaying spindle cells arranged in a storiform pattern in addition to vague fascicular and haphazard patterns with collagenous stroma.

A lung biopsy was performed; the specimen was composed of spindle cells arranged in storiform pattern, vague fascicular and haphazard patterns with collagenous stroma (Figure 4).

There was no evidence of necrosis in the specimen. Immunohistochemistry (IHC) was positive for Ki67 - low proliferative (≈5%). Our center has limited resources available and further IHC could not be performed. Although the essential World Health Organization (WHO) criteria for diagnosis of SFT could thus not be met, the pathology was suggestive and in conjunction with her recurrent bouts of hypoglycemia we diagnosed NICTH and DPS.

She commenced oral prednisone 30 mg/day which ameliorated her symptoms. She was subsequently referred to the thoracic surgical unit at a separate institution, where she was deemed not suitable for surgical resection at a multidisciplinary team (MDT) meeting. She received palliative care and died approximately six months after her first presentation to our institution.

## Discussion

The WHO defines SFT as a fibroblastic tumor characterized by a prominent, branching, thin-walled, dilated (staghorn) vasculature and NAB2-STAT6 gene rearrangement [11]. The WHO has outlined essential and desirable diagnosis criteria: essential comprises of spindled to ovoid cells arranged around a branching and hyalinized vasculature, variable stromal collagen deposition, CD34 and/or STAT6 expression by IHC. Desirable criteria (in selected cases) consist of the demonstration of NAB2-STAT6 gene fusion. The latest WHO classification no longer uses the terminology of "typical" and "malignant," but instead subdivides SFTs into three categories; benign (locally aggressive), NOS (rarely metastasizing), and malignant [11,12].

SFTs within the thorax may be classified by the site of origin; pleural, mediastinal, or pulmonary [2]. They predominantly occur within the pleura, with >80% originating from the visceral pleura [2]. SFTs may be asymptomatic or be associated with cough, chest pain, and dyspnea [5,13]. Occasionally, they present with symptoms of paraneoplastic syndromes, such as DPS or Pierre-Marie-Bamberg syndrome. DPS typically presents with hypoglycemia; hypokalemia is frequently discovered in laboratory investigation [14,15]. Neuroglycopenic and autonomic symptoms of hypoglycemia are both plausible; however, neuroglycopenic features, i.e., confusion, amnesia, dizziness, anxiety, loss of judgment, and psychosis are predominant [15,16]. Severe or protracted hypoglycemia may result in seizures or coma [15]. Acromegaloid changes have been reported in rare instances, with resolution subsequent to tumor resection [15].

The underlying mechanism of hypoglycemia in DPS is overproduction of IGF-II by the tumor; both mature IGF-II and incompletely processed forms which collectively referred to as high molecular weight IGF-II or big IGF-II [15,16]. IGF-II is a 7.5-kDa peptide, but NICTH produces a high molecular weight form in the 10-20-kDa range due to abnormal processing of the IGF-II precursor [14,15]. There are numerous assays for detecting abnormal IGF-II forms, hence IGF-II levels may be elevated or normal [14,15]. Elevated IGF-II suppresses insulin and growth hormone (GH), leading to low IGF-I levels [14]. IGF-II inhibits glucose production, enhances glucose uptake in skeletal muscle, and suppresses fatty acid release in adipocytes [14,16]. Thus, glycogenolysis, gluconeogenesis, and ketogenesis are inhibited [14-16]. Excessive stimulation of the insulin-like growth factor-1 receptor (IGF-IR) is postulated as the cause of acromegaloid features occasionally observed in patients with NICTH [16].

Evaluation of hypoglycemia is warranted in instances wherein Whipple’s triad is fulfilled - symptoms/signs of hypoglycemia, low plasma glucose concentration, and resolution of symptoms/signs upon reversal of hypoglycemia [17]. This consists of pursuing the possibility of exogenous insulin administration, critical illness, organ failure, and endogenous hyperinsulinism. Laboratory investigations include, but are not limited to insulin, proinsulin, β-hydroxybutyrate, C-peptide, liver and renal function panels, and cosyntropin stimulation tests. Hypoinsulinemic hypoglycemia in conjunction with low C-peptide and β-hydroxybutyrate levels suggests an insulin mimicking agent and necessitates measurement of IGF-I and IGF-II [15]. Total levels of IGF-II may be elevated or normal, thus the IGF-II:IGF-I ratio is considered [14,15]. The normal ratio is 3:1 whilst ratios of >10:1 are considered diagnostic of IGF-II-mediated hypoglycemia [14,15].

Following biochemical evidence of NICTH, radiologic investigations are warranted to localize the tumor [2,14]. Chest radiography is often the first-line modality, but cross-sectional imaging of the chest/abdomen/pelvis is reasonable as most SFTs occur in these locations. Radiographic features are nonspecific; chest radiography displays a well-circumscribed mass, frequently taller than wide and in contact with one or more pleural surfaces [2]. CT typically demonstrates a well-defined mass with heterogenous contrast enhancement due to excessive vasculature whilst MRI displays hypointensity on T1-weighted images and variable hypointensity to hyperintensity on T-2 weighted images (fibrous and cellular/myxoid areas, respectively) [2,11]. Larger or more aggressive cases may display increased heterogeneity because of regions of fibrosis, hemorrhage, myxoid/cystic degeneration, calcifications, and necrosis [2,11].

Imaging features are nonspecific, and diagnosis is thus dependent on histologic findings [2,3,11]. SFTs are variably cellular neoplasms characterized by haphazardly arranged spindled to ovoid cells with prominent staghorn vasculature [3,11]. Immunohistochemically they exhibit diffuse CD34, CD99, and B-cell lymphoma protein (BCL-2) expression [2,3]. NAB2-STAT6 gene fusions are pathognomonic for SFT but may be difficult to identify in routine cytogenetic studies; STAT6 IHC is a highly sensitive and specific surrogate for the NAB2-STAT6 gene fusion [11]. Risk stratification models are preferred over anatomical staging; the histological criteria devised by England et al. [5] in their 1989 review of 233 cases of SFTP is frequently used to distinguish benign and malignant SFTP but more recent multivariate risk models provide improved prognostication [11]. The most widely used model for metastatic risk, proposed by Demicco et al. [18], utilizes patient age, mitoses/mm^2^, and tumor size, with one variation also including the presence of necrosis [11], whilst the French Sarcoma Group (FSG) has proposed risk calculators incorporating clinical data, pathological features, and history of radiotherapy to predict survival, local recurrence, and distant metastatic risk [19].

Management of hypoglycemic episodes in patients with DPS consists of oral glucose and/or IV dextrose. Numerous medical modalities, consisting of increased caloric intake in conjunction with various pharmacotherapies, have been employed if hypoglycemic episodes persist, albeit described primarily in case reports and small-scale studies [7,14,20]. Glucocorticoid therapy is extensively documented as effective in preventing hypoglycemic episodes, however patient dependent dose titration is required [14,15]. Glucocorticoids act through a multiplicity of mechanisms stimulating hepatic gluconeogenesis, inhibiting peripheral glucose uptake, promotion of lipolysis, inhibiting transcription product of NAB2-STAT6, and reduction of IGF-II levels [14,15]. Recombinant growth hormone at supraphysiological doses reduces the occurrence of hypoglycemia by suppressing peripheral glucose uptake and stimulating gluconeogenesis and glycogenolysis [14,15]. Its use is limited by its adverse effects of fluid retention and orthostatic hypotension and a theoretical risk of stimulating the growth of the tumor itself [14,15]. Glucagon and somatostatin analogs have been used to little effect in multiple cases [14,15,20]. Additional possible treatment options in refractory cases include corn starch supplementation and the use of phosphatidylinositol 3 kinase (PI3K) inhibitors as the latter inhibits the signaling cascade of insulin receptors stimulated by enhanced IGF-II levels [21,22].

Total (R0) resection of the tumor remains the gold-standard definitive treatment of SFTP; in the event that complete resection is not possible, palliative tumor debulking is recommended [15]. Adjuvant therapy including chemoradiation and selective embolization has been reported effective, but there are no standardized regimens [20], stemming from a paucity of data. Perioperative radiotherapy combined with surgery is associated with reduced risk of local failures especially in patients with less favorable resection margins and in those with tumors with a high mitotic count [23]. Chemotherapy has been used in the setting of advanced or metastatic SFT; however, there are no prospective clinical trials. Much of the available evidence supports the use of anthracycline-based regimens as first-line while ifosfamide, dacarbazine, and trabectedin are further options [12,24]. Due to the highly angiogenic nature of SFTs, there is a recent focus on antiangiogenic therapies with a prospective single-arm phase II trial confirming the efficacy of pazopanib [25,26]. Recent studies have used the Response Evaluation Criteria in Solid Tumors (RECIST) and Choi criteria (originally developed to predict the response of gastrointestinal stromal tumors to imatinib) to evaluate therapeutic response to antiangiogenic agents [3,12]. However, these should be interpreted with caution [12].

Following resection with or without perioperative radiotherapy, patients at intermediate to high risk of recurrence should have radiological follow-up. Localized SFTs bear a good prognosis, yet 10-year recurrence can reach 25% [12]. The aforementioned risk assessment models should be applied to clinical practice. SFT recurrence is more frequent in incomplete resection, tumor seeding, and whether the initial tumor was extra-pleural [12].

## Conclusions

Patients who fulfill Whipple’s triad (symptoms/signs of hypoglycemia, low plasma glucose concentration, and resolution of symptoms/signs upon reversal of hypoglycemia) should undergo thorough investigations for the etiology of hypoglycemia. SFT, can in a small proportion of cases, result in NICTH. Initial management consists of resolving the acute episode of hypoglycemia. Total resection of the tumor is the definitive treatment, but surgery may not always be feasible, as in the case presented herein.
